# Cyberbullying Victimization and Adolescent Drinking Behavior: Deviant Peer Affiliation as a Mediator and Personal Growth Initiative as a Moderator

**DOI:** 10.3389/fpsyt.2020.572530

**Published:** 2020-09-24

**Authors:** Pei Chen, Mucheng Xin, Qi Xie, Chang Wei, Chengfu Yu, Xiong Gan, Xiaodong Xie, Wei Zhang

**Affiliations:** ^1^Department of Psychology and Research Center of Adolescent Psychology and Behavior, School of Education, Guangzhou University, Guangzhou, China; ^2^School of Psychology and Center for Studies of Psychological Application, South China Normal University, Guangzhou, China; ^3^School of Education and Sports Science, Yangtze University, Jingzhou, China; ^4^Human Resources Department, South China Normal University, Guangzhou, China

**Keywords:** cyberbullying, drinking behavior, deviant peer affiliation, personal growth initiative, adolescent

## Abstract

Research has demonstrated a robust positive association between cyberbullying victimization and adolescent drinking behavior; however, the mediating and moderating mechanisms underlying this relationship remain largely unexplored. Grounded in the social development model and person-environment interactions model, our study explored whether deviant peer affiliation mediated the relationship between cyberbullying victimization and adolescent drinking behavior and whether this mediating effect was moderated by personal growth initiative. A sample of 1,006 adolescents (*M_age_* = 13.16 years; *SD* = 0.67) anonymously completed self-report questionnaires. Structural equation modeling indicated that the positive association between cyberbullying victimization and drinking behavior was partly mediated by deviant peer affiliation for both girls and boys. Further, this mediating process was stronger for adolescents with low personal growth initiative than for those with high personal growth initiative. There were no significant gender differences for this moderating effect. These findings underline the importance of deviant peer affiliation and personal growth initiative in understanding how and when cyberbullying victimization impacts adolescent drinking behavior.

## Introduction

Cyberbullying victimization among adolescents represents a serious public health concern, as it has been shown to have an adverse impact on their psychological growth (1, 2). Cyberbullying victimization is a continuation of traditional bullying executed through electronic media, and includes aggressive online behavior, performed electronically by a group or individual repeatedly, over time to attack a victim without any self-protection abilities (3). Examples of cyberbullying victimization include repeatedly sending harassing or offensive messages to victims and publicly posting harmful information and content on the Internet (4, 5). Ample research has documented that there is a high prevalence of cyberbullying victimization among Chinese adolescents with rates that are increasing annually (6–8).

A considerable number of empirical studies have confirmed that cyberbullying victimization is a strong risk predictor for adolescent alcohol use (9–11). On the basis of general strain theory (12), high pressure circumstances (such as cyberbullying victimization) might increase the possibility of delinquent behavior (such as drinking behavior). In line with this theory, Ouyang et al. found a positive association between cyberbullying victimization and adolescent alcohol use (11). However, although these studies have documented the positive relationship between cyberbullying victimization and adolescent drinking behavior, the underlying mediating and moderating mechanisms remain largely unclear. Recent data indicate that more than 50% of adolescents use alcohol before graduating from high school (13). Drinking alcohol during adolescence is correlated with a variety of negative outcomes, including violent behavior and suicide (14). Given its high prevalence and detrimental effects on adolescent development, identifying the mechanisms underlying drinking behavior to enhance prevention programs and develop targeted interventions is essential.

### The Mediating Role of Deviant Peer Affiliation

Deviant peer affiliation is defined as the selective interaction with peers who engage in deviant behaviors, such as drinking, aggression and problematic Internet use (15). Based on the social development model (16), adolescents who are victimized may have an increased likelihood of interacting with deviant peers, which, in turn, may impact their development and behavior (e.g., increased rates of alcohol use). Specifically, the experience of cyberbullying victimization may interrupt the development of a social bond between youth and conventional society, subsequently increasing the likelihood of their association with delinquent peers. Recent research has documented that victimized adolescents increasingly affiliate with deviant peers (17, 18). For example, Jiang et al. found that adolescents experiencing peer victimization had a greater likelihood of affiliating with deviant peers (18). Further, cyber victimization has been associated with “traditional” forms of peer victimization and may be even more disruptive to adolescents’ lives than traditional victimization (19, 20).

Moreover, bonding with deviant peers might increase an adolescent’s chances of adopting beliefs and behaviors consistent with the norms of the deviant peer group, increasing the risk for alcohol use (16, 21). Compared to adolescents without deviant peer affiliation, adolescents affiliating with deviant peers have a greater likelihood of holding positive attitudes and views toward drinking and may develop drinking behavior easily through vicarious reinforcement. Mounting evidence has demonstrated that deviant peer affiliation was a notable predictor of adolescent drinking behavior (22–24). For instance, in a sample of 1,175 middle school students, Chen et al. found that adolescents who affiliated with deviant peers were more likely to engage in drinking (23).

Based on the social development model and existing research, we proposed the following hypothesis:

*Hypothesis 1*: Deviant peer affiliation would mediate the relationship between cyberbullying victimization and adolescent drinking behavior.

### The Moderating Role of Personal Growth Initiative

While cyberbullying victimization significantly contributes to adolescents’ drinking behavior *via* deviant peer affiliation, not all adolescents are equally likely to use alcohol after being cyberbullied. The person-environment interactions model (25) offers one theory to conceptualize the heterogeneity of adolescents’ drinking behavior, as it postulates that adolescent development results from the interaction between personal characteristics and environmental factors. Thus, in the present study, we examined the moderating role of personal growth initiative.

Personal growth initiative is defined as taking the initiative to engage in one’s own growth process, which comprises two significant aspects: active intra-individual change and intentional or purposeful behavior in nature (26, 27). Personal growth initiative may attenuate the mediating mechanism of deviant peer affiliation underlying the relationship between cyberbullying victimization and adolescent drinking behavior. Specifically, adolescents who report high levels of personal growth initiative would have greater abilities, and positive strategies to manage stressful situations, which could enhance their resilience and reduce the likelihood of delinquent behaviors such as deviant peer affiliation (28). Moreover, adolescents who affiliate with deviant peers may be protected by personal growth initiative, which could decrease their risk of problematic behaviors, such as alcohol use. For example, adolescents with a high level of personal growth initiative may have a strong bond with conventional society because of increased emotional support, making them less likely to use alcohol (11, 29). Therefore, adolescents with high levels of personal growth initiative are less likely to cope with cyberbullying victimization by affiliating with deviant peers and engaging in drinking behavior.

Although no published research to date has examined the role of personal growth initiatives in the indirect relation between cyberbullying victimization and adolescent alcohol use, some research has provided support for the moderating role of personal growth initiative. For example, Robitschek et al. stressed that personal growth initiative played a protective role in the recovery process of psychopathology (30). Similarly, personal growth initiative moderated the relationship between stress and student psychological adjustment (28). These findings are consistent with the risk-buffering model (31), which posits personal assets can buffer or mitigate the deleterious effects of environmental risks on adolescent development. Therefore, based on previous research and theories, we hypothesized the following:

*Hypothesis 2*: Adolescent personal growth initiative would moderate the mediating mechanism of deviant peer affiliation underlying the association between cyberbullying victimization and adolescent drinking behavior. The mediation process would be significant among those adolescents with low personal growth initiative, but weak among those with high personal growth initiative.

### The Present Study

**Figure 1:**
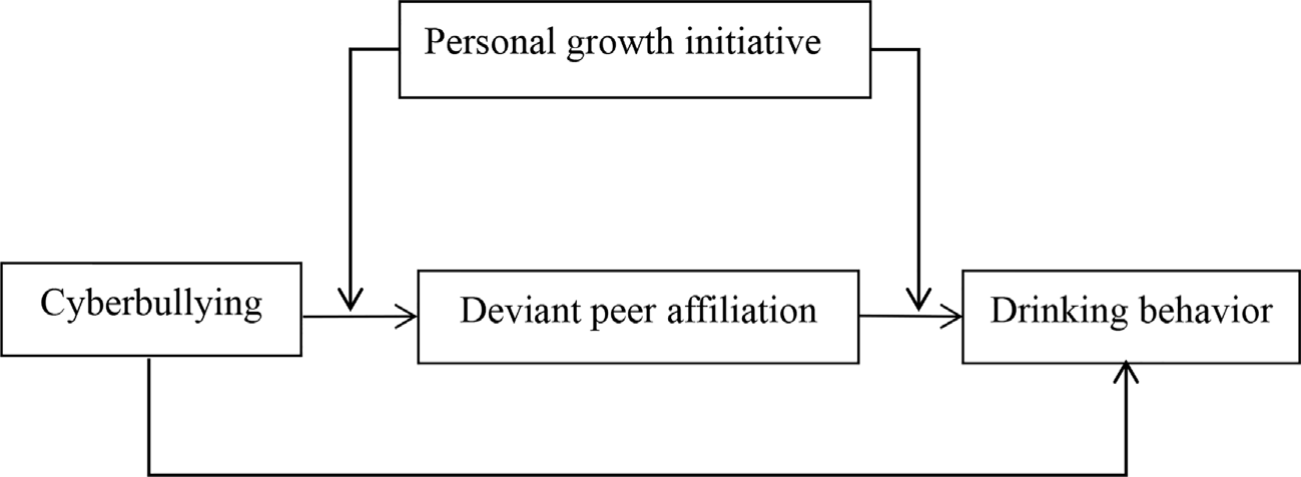
The proposed moderated mediation model.

Grounded in the social development model (16) and person-environment interaction model (25), this study aimed to examine deviant peer affiliation as a mediator and personal growth initiative as a moderator to account for how and when cyberbullying victimization impacts adolescent drinking behavior. Specifically, we sought to test whether deviant peer affiliation mediated the direct relationship between cyberbullying victimization and adolescent drinking behavior and whether personal growth initiative moderated this indirect association. **Figure 1** illustrates the proposed research model.

## Method

### Participants

In this study, participants were recruited from three middle schools in Guangdong province, southern China, through stratified and random cluster sampling. The sample was stratified by city size (large, medium, and small cities). The data was collected during September 2019. Random cluster sampling was used to select three grade 7 classes and three grade 8 classes of an ordinary middle school from each of city. A total of 1,006 adolescents (485 male and 521 female) ranging from 12 to 15 years old (*M*_age_ = 13.16 years, *SD* = 0.67) participated.

### Measures

#### Cyberbullying Victimization

Cyberbullying victimization was assessed with the Cyberbullying Victimization Scale (32). Participants indicated how many times they had experienced cyberbullying victimization in the past 6 months (e.g., “Some people have spread rumors about me and bad-mouthed me online”) on a 4-point scale ranging from 1 = *never* to 4 = *five or more times*. Scores for all items were averaged, with higher scores representing greater instance of cyberbullying victimization. In this study, the measure demonstrated good reliability (*α* = 0.82).

#### Deviant Peer Affiliation

Deviant peer affiliation was assessed with a 12-item Chinese version questionnaire (33). Participants indicated how many of their friends had engaged in deviant behaviors in the past 6 months (e.g., “How many of your friends have cheated on exams?”) on a 5-point scale ranging from 1 = *never* to 5 = *six or more times*. Scores for all 12 items were averaged, with higher scores representing greater deviant peer affiliation. In this study, the measure demonstrated good reliability (*α* = 0.81).

#### Personal Growth Initiative

Personal growth initiative was assessed using the Personal Growth Initiative Scale-II, developed by Robitschek et al. (34). Participants indicated how true each item was of them (e.g., “I can grasp every opportunity for growth”) on a 5-point Likert scale ranging from 1 = *strongly disagree* to 6 = *strongly agree*. Scores for all 16 items were averaged, with higher scores representing higher personal growth initiative. In this study, the measure demonstrated good reliability (*α* = 0.95).

#### Drinking Behavior

Participants reported the average number of times per month they had used alcohol (including beer, wine, and hard liquor) in the past 6 months using a 6-point scale from 1 = *never* to 6 = 8 *or more times*. Higher scores indicated higher levels of drinking behavior. This instrument has demonstrated good validity in previous studies (18, 23, 35). Given studies have shown that drinking behavior significant associate with depression (36), this study use depression as the indicator of criterion-related validity. Depression was assessed using the Center for Epidemiological Studies Depression Scale (37), and demonstrated good reliability in this study (*α* = 0.88). In this study, drinking behavior was positively correlated with depression (*r* = 0.21, *p* < 0.01), indicating this measure has good criterion validity.

#### Control Variables

Previous literature has found that gender, age, and sensation seeking to be significantly related to adolescent drinking behavior (38, 39); therefore, we controlled for these variables in the present study. Gender was dummy coded such that 1 = *male* and 0 = *female*. The 4-item sensation seeking, subscale of the Impulsive Behavior Scale was used to measure participants’ sensation seeking (40). Participants were asked to assess their sensation seeking tendencies (e.g., “I like the feeling of adventure”) on a 4-point scale, ranging from 1= *strongly agree* to 4= *strongly disagree*. Scores were calculated using an average of the four items, with higher scores reflecting higher levels of sensation seeking. This instrument has demonstrated good reliability in previous studies (39, 41). In this study, the measure demonstrated good reliability (*α* =0.73).

### Procedure

This research used a collective test method, taking the class as a unit to investigate the students using self-report questionnaires. Before the research was begun, we obtained approval from the Academic Ethics Review Committee of the School of Education, Guangzhou University. Additionally, the adolescent participants and their guardians and school administrators provided informed assent and informed consent, respectively, before participants completed all questionnaires with support from trained research assistants who were students in psychology.

### Statistical Analyses

For our preliminary analyses in the current study, we conducted reliability analysis and descriptive analysis using SPSS 25.0 software. Next, we conducted structural equation modeling by using full-information maximum likelihood estimation. Then, we tested the mediation and moderation mechanisms by using the bootstrap method with 1,000 replications of the data in Mplus 7.1 (42). Based on statisticians’ suggestions (43), three indices (include *χ*²*/df*, CFI, and RMSEA) were used to indicate model goodness of fit. The model fit is considered acceptable when *χ*²*/df* < 3, CFI > 0.95, and RMSEA < 0.06 (43). Participants’ gender, age, and sensation seeking tendencies were included as covariates in the above analysis.

## Results

### Preliminary Analyses

The means, standard deviations, and correlation coefficients for all variables of the current study are displayed in **Table 1**. The results showed that cyberbullying victimization were positively correlated to deviant peer affiliation and drinking behavior. Personal growth initiative was negatively associated with drinking behavior. Moreover, deviant peer affiliation was positively correlated to drinking behavior.

**Table 1:** Descriptive statistics and correlations for all variables.

Variables	1	2	3	4	5	6	7
1. Gender	1.00						
2. Age	0.06	1.00					
3. SS	0.05	−0.06	1.00				
4. CV	0.02	0.00	0.14**	1.00			
5. PGI	0.02	−0.02	−0.01	−0.09**	1.00		
6. DPA	0.06*	−0.11**	0.19**	0.40**	−0.08*	1.00	
7. DB	−0.02	0.05	0.10**	0.18**	−0.11**	0.18**	1.00
*Range*	0–1	12–15	1–4	1–4	1–5	1–5	1–6
*Mean*	0.48	13.16	2.00	1.13	3.54	1.19	1.07
*SD*	0.50	0.67	0.67	0.20	0.73	0.31	0.30

Gender were dummy coded such that 1= male, 0 = female. SS, sensation seeking; CV, cyberbullying victimization; PGI, personal growth initiative; DPA, deviant peer affiliation; DB, drinking behavior. *p < 0.05, **p < 0.01.

### The Mediating Effect of Deviant Peer Affiliation

**Figure 2:**
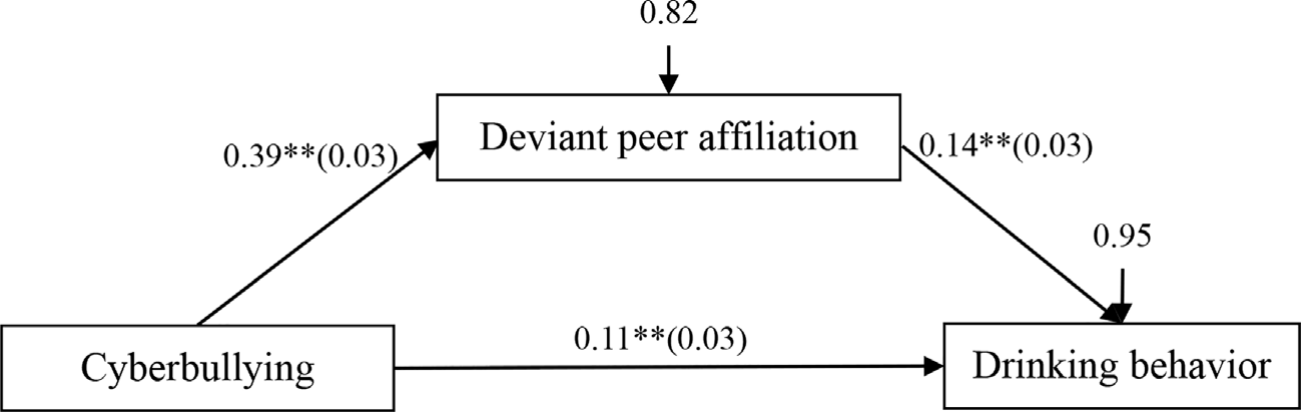
Model of the mediating role of deviant peer affiliation between cyberbullying victimization and drinking behavior. ** p < 0.01.

**Figure 3:**
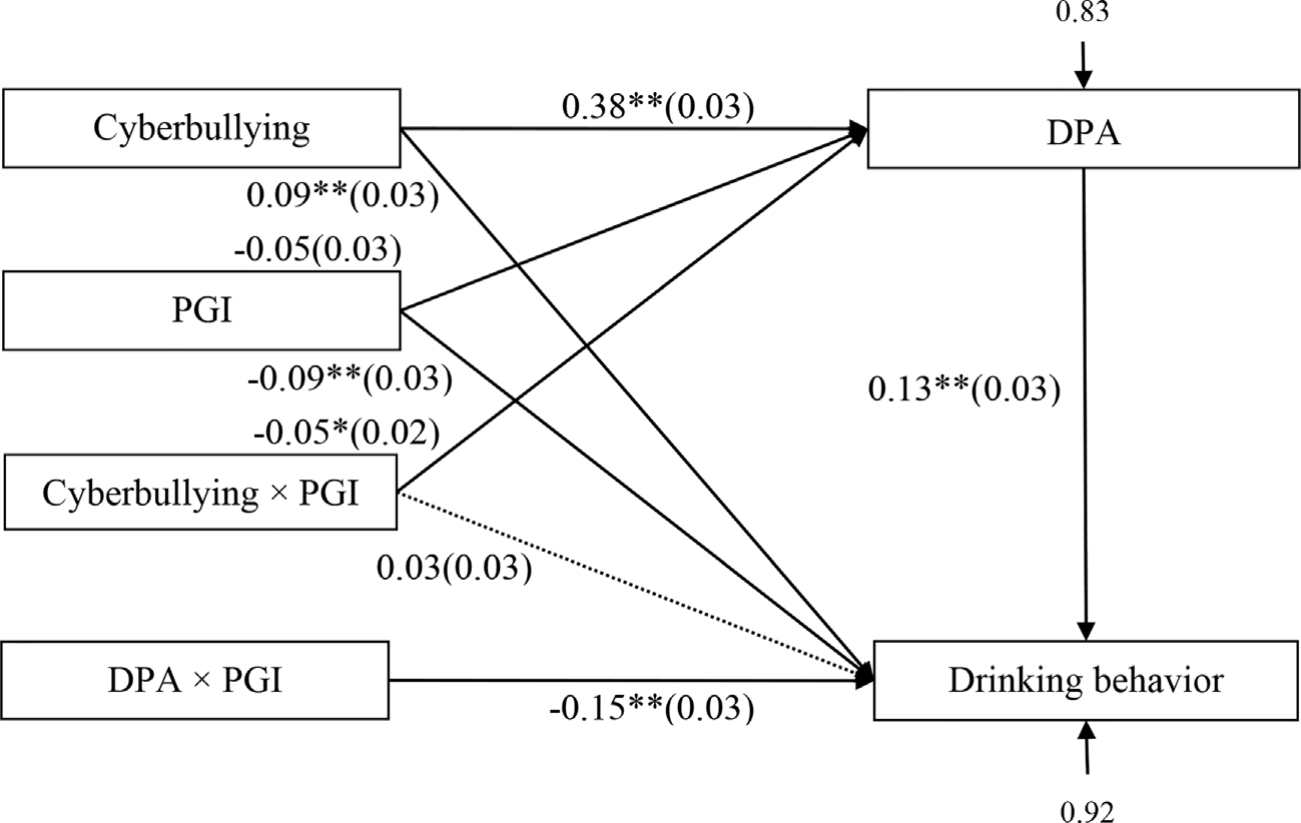
Model of the moderating role of personal growth initiative on the indirect relationship between cyberbullying victimization and drinking behavior. PGI, personal growth initiative; DPA, deviant peer affiliation. * p < 0.05, ** p < 0.01.

The mediation model represented in **Figure 2** revealed an acceptable fit to the data: *χ*^2^/*df* = 1.57, CFI = 0.99, RMSEA = 0.02. The results are displayed in **Figure 3**. cyberbullying victimization was positively associated with deviant peer affiliation (*β* = 0.39, *SE* = 0.03, *t* = 13.47, *p* < 0.01, 95% CI [0.34, 0.45]), and deviant peer affiliation was positively associated with drinking behavior (*β* = 0.14, *SE* = 0.01, *t* = 4.05, *p* < 0.01, 95% CI [0.07, 0.21]). Moreover, residual effect of cyberbullying victimization on drinking behavior was significant (*β* = 0.11, *SE* = 0.03, *t* = 3.31, *p* < 0.01, 95% CI [0.05, 0.18]). Bootstrapping analyses indicated that deviant peer affiliation significantly mediated the relation between cyberbullying victimization and adolescent drinking behavior (indirect effect = 0.0548, *SE* = 0.0224, 95% CI [0.0187, 0.1077]).

### Testing for Moderated Mediation

The moderated mediation model represented in **Figure 3** revealed a good fit to the data: *χ*^2^/*df* = 2.63, CFI = 0.99, RMSEA = 0.04. The bias-corrected percentile bootstrap results indicated that the indirect effect of cyberbullying victimization on adolescent drinking behavior through deviant peer affiliation was moderated by personal growth initiative. Specifically, personal growth initiative moderated the association between cyberbullying victimization and deviant peer affiliation (*β* = −0.05, *SE* = 0.02, *t* = −2.07, *p* < 0.05, 95% CI [−0.10, −0.003]), and the association between deviant peer affiliation and drinking behavior (*β* = −0.15, *SE* = 0.03, *t* = −4.62, *p* < 0.01, 95% CI [−0.21, −0.08]).

**Figure 4:**
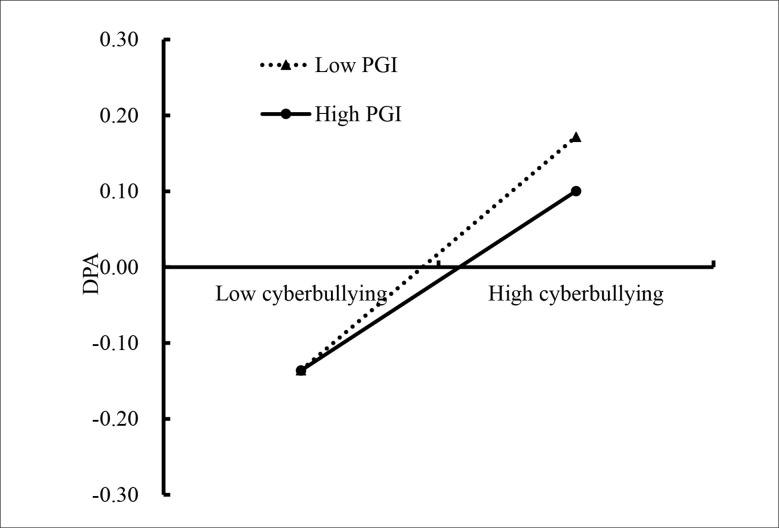
Deviant peer affiliation among adolescents as a function of cyberbullying victimization and personal growth initiative. CV, cyberbullying victimization; PGI, personal growth initiative; DPA, deviant peer affiliation.

**Figure 5:**
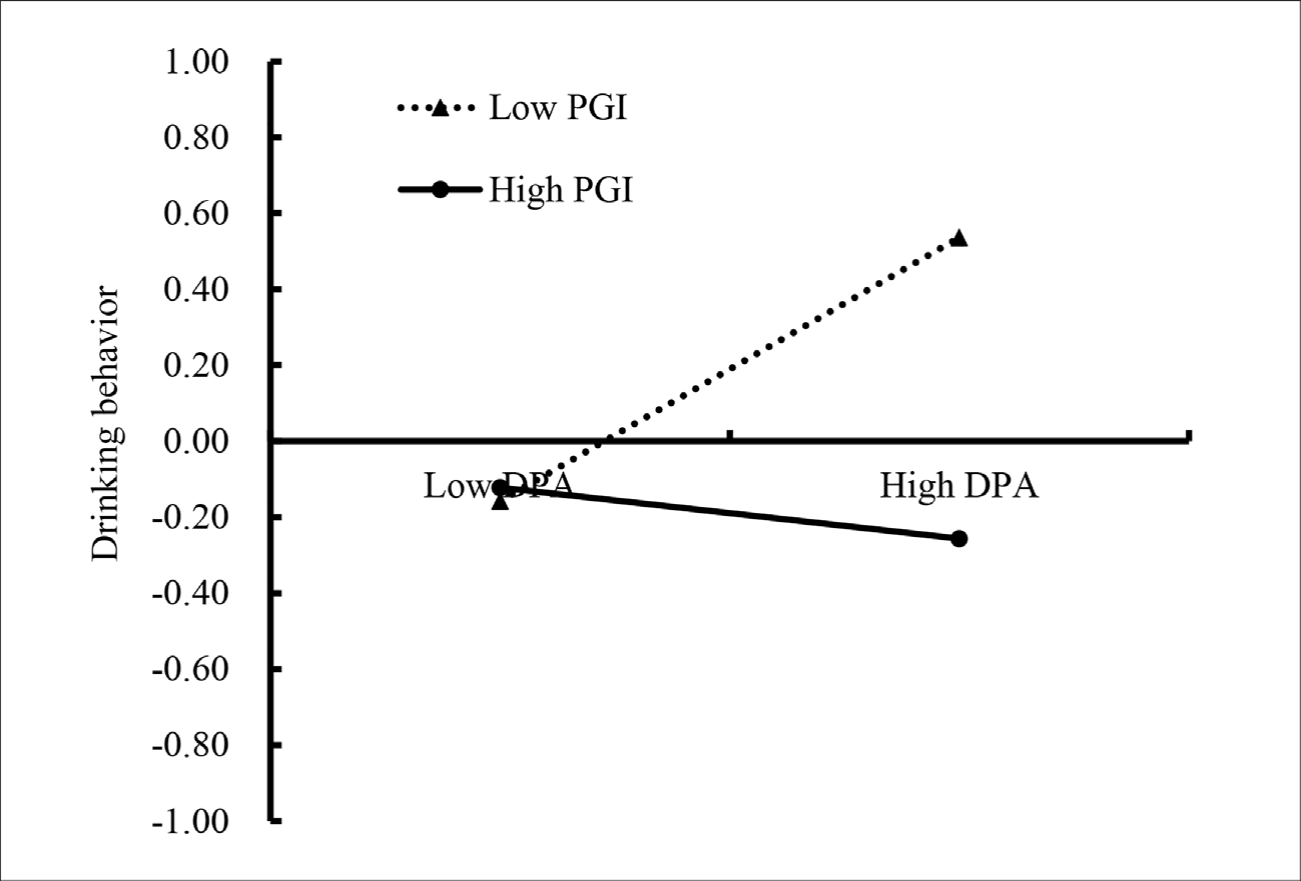
Drinking behavior among adolescents as a function of deviant peer affiliation and personal growth initiative. PGI, personal growth initiative; DPA, deviant peer affiliation.

We conducted two simple slopes test, and as depicted in **Figures 4**, **5**. **Figure 4** illustrates deviant peer affiliation among adolescents as a function of cyberbullying victimization and personal growth initiative. The results showed that the positive association between cyberbullying victimization and deviant peer affiliation was much stronger for adolescents with lower personal growth initiative (*β* = 0.43, *SE* = 0.04, *t* = 12.23, *p* < 0.01, 95% CI [0.36, 0.50]) compared to adolescents with higher personal growth initiative (*β* = 0.33, *SE* = 0.04, *t* = 7.94, *p* < 0.01, 95% CI [0.25, 0.41]). Moreover, **Figure 5** illustrates drinking behavior among adolescents as a function of deviant peer affiliation and personal growth initiative. The results showed that deviant peer affiliation is significantly associated with drinking behavior among adolescents with higher personal growth initiative (1*SD* above the mean; *β* = −0.18, *SE* = 0.04, *t* = −3.95, *p* < 0.01, 95% CI [−0.26, −0.09]). However, this link between deviant peer affiliation and drinking behavior was not significant among adolescents with lower personal growth initiative (1*SD* below the mean; *β* = −0.04, *SE* = 0.04, *t* = −1.02, *p* > 0.05, 95% CI [−0.13, 0.04]).

Moreover, personal growth initiative had a significant negative association with deviant peer affiliation (*β* = −0.12, *SE* = 0.03, *t* = −3.99, *p* < 0.01, 95%CI [−0.17, −0.06]) and a significant positive relationship with drinking behavior (*β* = 0.09, *SE* = 0.03, *t* = 2.87, *p* < 0.01, 95%CI [0.03, 0.15]). However, the interaction between cyberbullying victimization and personal growth initiative in impacting deviant peer affiliation (*β* = −0.01, *SE* = 0.03, *t* = −0.39, *p* > 0.05, 95%CI [−0.06, 0.04]) and drinking behavior (*β* = 0.01, *SE* = 0.03, *t* = 0.44, *p* > 0.05, 95%CI [−0.04, 0.07]) was not significant.

Moreover, the indirect link between cyberbullying victimization and drinking behavior *via* deviant peer affiliation was significant for adolescents with higher personal growth initiative (indirect effect = 0.0173, *SE* = 0.01, 95% CI [0.0008, 0.0396]). However, this indirect link was nonsignificant for those with lower personal growth initiative (indirect effect = 0.0034, *SE* = 0.0047, 95% CI [−0.0016, 0.0189]). Therefore, the mediating effect of deviant peer affiliation between cyberbullying victimization and adolescent drinking behavior was moderated by personal growth initiative.

## Discussion

Inspired by the social development model (16) and the person-environment interaction model (25), this study examined the mediating influence of deviant peer affiliation on the association between cyberbullying victimization and adolescent drinking behavior. Further, we investigated the moderating role of personal growth initiative on this indirect relationship. Our findings contribute to current understanding in the field of the mechanisms that influence the effect of cyberbullying victimization on adolescent drinking behavior.

### The Mediating Effect of Deviant Peer Affiliation

Consistent with Hypothesis 1, cyberbullying victimization was related to adolescent drinking behavior *via* deviant peer affiliation. Thus, deviant peer affiliation was not only the result of cyberbullying victimization but also served as a catalyst for adolescent alcohol use. These findings are in line with the social development model (16), which suggests that adolescents experiencing cyberbullying victimization would have an increased likelihood of affiliating with deviant peers, which, in turn, increases their risk of engaging in drinking behavior.

Specifically, when adolescents experience cyberbullying victimization, they are more likely to affiliate with deviant peers. Cyberbullying victimization might interrupt the development of a social bond of belief between youth and conventional society, which would increase the likelihood they would associate with delinquent peers (21). When adolescents affiliate with deviant peers, they are more likely to use alcohol (23, 24). Under peer pressure and vicarious reinforcements, adolescents affiliating with deviant peers have increased opportunities to develop positive attitudes and values regarding alcohol use. Thus, they more easily engage in drinking behaviors than adolescents who do not associate with undesirable peers (18, 21). These findings are consistent with the previous research demonstrating that deviant peer affiliation acts as a critical mediator linking victimization to adolescent drinking behavior (18, 44, 45). However, existing research has focused primarily on traditional forms of bullying (e.g., peer victimization). To our knowledge, our study is the first to indicate that deviant peer affiliation could have a mediating role in the relationship between cyberbullying victimization and drinking behavior among adolescents. Moreover, cyberbullying victimization exerts a direct influence over adolescent drinking behavior; therefore, adolescents who are cyberbullied may drink alcohol even without the mediator of deviant peer affiliation. Our findings suggest that the adverse impact of cyberbullying victimization should not be underestimated, and we need to pay more attention to the intervention and prevention of cyberbullying victimization.

### The Moderating Role of Personal Growth Initiative

Consistent with Hypothesis 2, this study also found that personal growth initiative moderated the risk of cyberbullying victimization on adolescent drinking behavior *via* deviant peer affiliation. Specifically, the indirect link between cyberbullying victimization and alcohol use was stronger for adolescents with low personal growth initiative than for those with high personal growth initiative. This finding is consistent with the person-environment interactions model (25) and existing research that has focused primarily on the role of personal growth initiative in improving mental health and adaptability (30, 46). Our findings extend previous research and are the first to identify the attenuating influence of personal growth initiative in the indirect relationship between cyberbullying victimization and adolescent alcohol use. Further, these results suggest that personal growth initiative serves as a protective factor against the indirect adverse impact of cyberbullying victimization on adolescent drinking behavior.

First, personal growth initiative attenuated the relationship between cyberbullying victimization and deviant peer affiliation. Per the risk-buffering model (31), psychological assets, such as personal growth initiative, can mitigate the adverse effects of environmental risk on adolescents. While cyberbullying victimization may have deleterious effects on adolescent development, for those adolescents with high levels of personal growth initiative, cyberbullying victimization may be viewed as a challenge for their personal growth and confidence in their own ability to cope successfully with challenges (27, 34). Since they have a greater ability to manage stress (28), adolescents with high levels of personal growth initiative are less likely to affiliate with deviant peers when attempting to cope with cyberbullying victimization.

Second, personal growth initiative buffered the risk of deviant peer affiliation on adolescent drinking behavior. Concretely, compared with adolescents with higher levels of personal growth initiative, adolescents with lower levels engaged in more drinking behaviors after affiliating with deviant peers. This moderating effect may be result from adolescents who have high levels of personal growth initiative also having an increased capacity for adjusting to the environment and the ability for self-improvement, thus, deviant peer affiliation may be a normative aspect of adjusting to their environment (47, 48). Further, adolescents with high levels of personal growth initiative may be strongly connected to their social environment due to receiving increased emotional support (11, 29). As they are involved with multiple levels of their environment, deviant peers may not be their only source of learning values and behaviors (49). Personal growth initiative may be powerful enough to resist the adverse influence of deviant peer affiliation, which can decrease adolescents’ risk of engaging in drinking behavior. In addition, adolescents with high levels of personal growth initiative tend to utilize positive coping strategies and may be aware of the danger of alcohol (50). Hence, personal growth initiative can moderate the role of deviant peer affiliation on drinking behavior.

Overall, this study was the first to explore the mediating role of deviant peer affiliation and the moderating role of personal growth initiative in the relationship between cyberbullying victimization and adolescent drinking behavior, which provides important insights regarding the effects of cyberbullying victimization on adolescent drinking behavior. Our findings indicate that cyberbullying victimization interacts with other factors, including deviant peer affiliation and personal growth initiative to contribute to the development of adolescent drinking behavior. These results have critical implications for future research and practice.

### Study Limitations and Future Directions

Although this study clarifies the mediating and moderating mechanisms of how cyberbullying victimization leads to adolescent drinking behavior, several limitations should be noted. First, this study used a cross-sectional design, making it impossible to understand the temporal order of the processes and factors; therefore, causal influences cannot be determined. In the future, studies should adopt longitudinal and prospective designs and collect data from multiple timepoints. Second, this study relied solely on the self-reports of adolescents for data, which may result in some common method bias (51). Future research must use multi-method designs and collect multi-source data to decrease common method variance. Third, this study identified one significant pathway from cyberbullying victimization to adolescent drinking behavior. Future research could consider other potential pathways (e.g., peer rejection and school disengagement). Additionally, this study first examined the protective role of personal growth initiative in the relationship between cyberbullying victimization, deviant peer affiliation, and alcohol use. Results indicated that personal growth initiative might act as a protective factor in adolescent socialization. Future studies should consider the moderating influence of personal growth initiative on relationships between other stressful situations and problem behaviors (e.g., exposure to community violence and problematic Internet use).

### Practical Implications

This study has meaningful implications for the prevention and intervention of adolescent drinking behavior. First, our findings suggest that reducing cyberbullying victimization may be a feasible method of preventing alcohol use in adolescents. Therefore, it seems to be important to provide cyberbullying victimization victims with emotion regulation training and psychological counseling to reduce the distress and other negative effects caused by cyberbullying victimization. Second, the results document that deviant peer affiliation may mediate the relationship between cyberbullying victimization and adolescent drinking behavior. Thus, it is important to provide cyberbullying victimization victims with social support (i.e., opportunities to socialize with peers) to reduce the likelihood they will affiliate with deviant peers. Parents and educators should also increase their behavioral monitoring of adolescents to decrease deviant peer affiliation. Third, the mediating role of deviant peer affiliation was moderated by personal growth initiative, which provided evidence that personal growth initiative might play a crucial role in individual development. Therefore, it may be necessary to increase personal growth initiative by conducting intentional growth training in regular school courses and further improving the effectiveness of related interventions (52). Finally, our integrated model suggests that both environmental resources (e.g., the cyber environment and deviant peers) and personal assets (e.g., personal growth initiative) should be considered when identifying methods for reducing alcohol use among adolescents.

## Data Availability Statement

The raw data supporting the conclusions of this article will be made available by the corresponding authors, without undue reservation.

## Ethics Statement

The studies involving human participants were reviewed and approved by the Academic Ethics Review Committee of the School of Education, Guangzhou University. Written informed consent to participate in this study was provided by the participants’ legal guardian/next of kin.

## Author Contributions

PC, CW, CY, and WZ conceived and designed the research. PC, CW, CY, and WZ performed the research. CY analyzed the data. PC, MX, QX, CW, CY, XG, XX, and WZ contributed to the writing and the revise of the manuscript. All authors contributed to the article and approved the submitted version.

## Funding

This study was supported by the 13th Five-Year Plan for the Development of Philosophy and Social Sciences of Guangdong Province (GD16XXL07) and National Natural Science Foundation of China (31671154).

## Conflict of Interest

The authors declare that the research was conducted in the absence of any commercial or financial relationships that could be construed as a potential conflict of interest.
